# Medical Student Utilization of a Novel Web-Based Platform (Psy-Q) for Question-Based Learning in Psychiatry: Pilot Questionnaire Study

**DOI:** 10.2196/18340

**Published:** 2020-07-06

**Authors:** John Torous, Zev Nakamura, Jordan Rosen, Pochu Ho, Christine Pelic, Larkin Elderon Kao, David Kasick, Joseph Witowsky, Fremonta Meyer

**Affiliations:** 1 Beth Israel Deaconess Medical Center Boston, MA United States; 2 University of North Carolina Chapel Hill, NC United States; 3 Yale School of Medicine New Haven, CT United States; 4 Ralph H Johnson Veterans Administration Medical Center Charleston, SC United States; 5 Boston University School of Medicine Boston, MA United States; 6 VA Boston Healthcare System Boston, MA United States; 7 Ohio State University Wexner Medical Center Columbus, OH United States; 8 Brigham & Women’s Hospital Boston, MA United States

**Keywords:** medical students, education, psychiatry

## Abstract

**Background:**

Medical students are turning to new and expanding web-based resources for learning during their psychiatry clerkships; however, there have not been concomitant efforts by educators to utilize web-based tools to promote innovative teaching.

**Objective:**

Utilizing a free learning platform (Psy-Q) created by our team, we sought to explore how digital technology may engage medical student learners, promote colearning between educators and medical students, and support sustainability of web-based platforms through crowdsourcing.

**Methods:**

Between 2017 and 2019, seven medical schools offered access to the platform during medical students’ psychiatry clerkships. Use of the web-based platform was voluntary and not monitored or related to clerkship evaluation. Medical students completed a paper and pencil assessment of the platform at the end of their clerkship. Anonymous and aggregated website use data were gathered in accordance with institutional review board approval.

**Results:**

A total of 203 medical students across seven distinct psychiatry clerkships completed the survey. Of these students, 123 (60.6%) reported using the platform and reported accessing a mean of 45 questions. The most common device used to access the platform was a laptop and the second most common was a smartphone. The most common location to access the platform was home and the second most common was the hospital. Although few students contributed new questions, website utilization data suggested that all rated the quality and difficulty of the questions. Higher quality questions were medical students’ main suggestion for further improvement.

**Conclusions:**

Our results suggest the feasibility and potential of educator- and learner-created web-based platforms to augment psychiatry education and develop relevant accessible resources in the digital sphere. Future work should focus on measuring objective educational outcomes of question taking and writing, as well as optimizing technology and exploring sustainable trainee-faculty partnership models for the creation and curation of content.

## Introduction

Although web-based learning resources for medical student education in psychiatry offer enormous potential benefits, there persists a “digital divide” between learners and psychiatry educators. Increasingly, medical students are foregoing printed material, such as books, in favor of web-based and digital resources of more heterogeneous quality. Our team previously created a free web-based platform Psy-Q [1,2] in the hopes of bridging this digital divide and engaging both students and educators in collaborative learning with digital tools. In this follow-up report, we assessed medical student uptake and satisfaction with the platform across seven psychiatry clerkships.

There is a clear unmet need for high-quality web-based resources in psychiatry education. Survey research involving medical students rotating on psychiatry clerkships suggests that 90% want more educational smartphone apps [3] and that only a minority currently use printed material like text books or review books [4]. Increasingly, medical students are utilizing question banks [5] and are even creating their own in some instances [6]. Yet, many medical student question banks are expensive [7], and their content is often of unknown quality. Psy-Q offers a free, mobile-compatible, web-based question bank having high-quality questions, with each question requiring a reference from the medical literature and vetting by educators.

Psychiatry educators have also recently realized the potential of technology. As Hilty and DeJong aptly write in Academic Psychiatry, “the profession has to consider new applications of technology as instrumental, rather than supplemental, to practice and teaching” [8]. E-learning platforms can offer flexible tools to psychiatry educators but are most powerful when utilized for collaboration and engagement, rather than as static resources [9]. Although it can be useful for psychiatry educators to be aware of popular web-based resources [10], cocreating such resources with learners and educators may offer a more engaging and higher quality alternative. In designing the Psy-Q platform, we sought to remove technical barriers for educators to create content on a multimedia web-based platform and facilitate learning directly with their students.

Realizing the challenges faced by web-based question banks, we created the platform to foster collaboration and curation [1]. Psy-Q allows students and educators to easily submit their own questions, but all student questions must be approved by an educator who can send the question back to the student for rounds of revision. In teaching students how to write questions, the platform offers didactic benefits not present in traditional question banks. As a further quality measure, students and educators are able to rate questions so that poor-quality questions are flagged for educators to review and potentially remove.

Understanding medical students’ use and perception of the Psy-Q platform is important to assess web-based resource utilization patterns and ultimately improve the quality of learner and educator collaboration. Therefore, we designed a survey to capture on what devices and in what settings medical students reported using the platform, as well as their engagement in taking and creating questions. At the time the survey was administered, the website contained approximately 170 questions collaboratively written by a combination of psychiatry trainees and faculty. We hypothesized that a majority of students would access the platform, use the platform most often on smartphone devices at home, and use the platform more for responding to questions than for writing original questions.

## Methods

Medical students completing their core psychiatry clerkship were introduced to the platform via flyers or a brief orientation by a faculty member. It was strongly emphasized that use of the platform and participation in the follow-up survey were both entirely voluntary and would not impact clerkship evaluations. At the conclusion of the clerkship, an 11-item survey (Multimedia Appendix 1) was administered to the students. Institutional review board exemption was obtained by Harvard Medical School followed by all other sites.

The study was conducted at a total of seven medical schools (Harvard Medical School, University of Virginia, Yale School of Medicine, University of North Carolina, Medical University of South Carolina, Ohio State University, and Boston University School of Medicine) and eight unique psychiatry clerkships (including two separate clerkship sites within Harvard Medical School; data were pooled into a single site for the purpose of analysis by the medical school) that each collected data for 6 months between 2017 and 2019. The study authors FM, DK, CP, ZN, LK, PH, and JR were consultation-liaison psychiatry rotation directors or supervisors at seven of the eight study sites.

**Figure 1:**
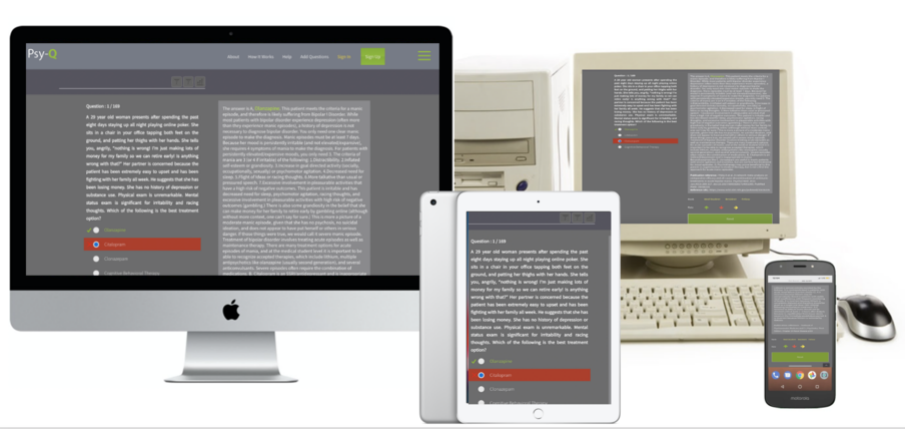
Screenshots of Psy-Q on multiple platforms. Multiple platforms can be used to access Psy-Q.

Analysis was conducted using descriptive statistics (frequencies or percentages). The associations between study variables were assessed using chi-square or Fisher exact tests. All data analyses were conducted with R using the *dplyr* package (version 3.5.3, R Foundation for Statistical Computing). Screenshots of the Psy-Q platform as accessed from a computer and mobile phone are shown in Figure 1.

## Results

Overall, 203 students across seven distinct psychiatry clerkships completed the survey. Sites differed in the number of students participating in the survey, the number of students accessing the Psy-Q platform, and the average number of questions completed (Table 1).

Of 203 students, 146 (71.9%) reported utilizing mainly web-based resources for psychiatry learning during their clerkship. Additionally, of 203 students, 123 (60.6%) reported accessing the platform, with students reporting having responded to an average of 45 questions each (total of 5535). Based on anonymous website use data involving both students in the study and those using the website outside of the study at other sites, 9126 total questions were taken, suggesting that students outside of the study may have accessed and utilized the Psy-Q platform as well. Additionally, based on the anonymous website data, the mean number of questions taken across all users was 42, similar to that reported by students in the study. Although the study does not enable direct linkage of individual medical students to their web-based activity on the Psy-Q platform, the data offer a window into how the platform is utilized.

Anonymous website data indicated that users offered 735 “thumbs up” votes, 209 neutral votes, and 126 “thumbs down” votes regarding their opinions of both the questions and the subsequent answer explanations. Of 9126 questions, 6278 (68.79%) were correctly answered on the first attempt. The average user spent 34 minutes taking questions, and the most common platform for accessing the Psy-Q website was a personal computer.

According to self-reports, only two questions were added to the website during the study. The most commonly used combination involved a laptop at home, although students could use multiple devices to access the platform from multiple locations, making specific assessment challenging. Of the 121 students who answered the question about the most commonly used device, 74 (61.2%) reported accessing the platform via a laptop, 21 (17.4%) reported accessing via a smartphone, 16 (13.2%) reported accessing via a tablet, and 10 (8.3%) reported accessing via a desktop computer. Of the 117 students who answered the question about location, 72 (61.5%) reported home as the site of access, 32 (27.4%) reported hospital, 9 (7.7%) reported library, and 5 (4.3%) reported transportation during the commute to school.

Of the 123 students who accessed the platform, the mean rating of usefulness was 6.7 out of 10, with 10 being most helpful. The mean utility rating did not significantly differ by the number of questions taken (*P*=.17), study site (*P*=.41), device (*P*=.09), or access setting (*P*=.52). Of the 123 respondents, 109 (88.6%) reported that they would recommend the platform to other students.

Of the 123 students who responded to the question about areas for possible improvement, 28 (22.8%) cited quality of the questions and answers, followed by ease of use of the platform (20 students, 16.3%), difficulty of the questions (16 students, 13.0%), and esthetics (11 students, 8.9%). The survey did not assess potential concerns with question volume. There was no association between any single area of improvement and overall satisfaction with the platform.

**Table 1:** Data from the seven study sites, their engagement with the survey, and reported use of the Psy-Q platform.

Site	Clerkship length (weeks)	Shelf exam (yes/no)	Students partaking in the study and response rate if available, n (%)	Students reporting accessing the Psy-Q platform, n (%)	Average number of reported questions taken
1	4	Yes	24 (80%)	17 (70%)	68
2	4	Yes	42 (93%)	32 (76%)	48
3	6	No	28	17 (60%)	45
4	5	Yes	66 (79%)	38 (58%)	44
5	6	Yes	27	12 (44%)	18
6	6	Yes	11 (100%)	4 (36%)	43
7	16 (combined with neurology and internal medicine)	Yes	5 (30%)	5 (100%)	17

## Discussion

### Principal Findings

Our results indicate that medical students across seven distinct psychiatry clerkships found the Psy-Q platform useful (61% utilization rate; mean rating of 6.7/10) and accessed over 5500 questions during the study period. Notably, this was strongly framed as a voluntary resource; psychiatry clerkships included many other required assignments and educational activities during the brief study period, as well as several other question bank resources from which to choose (eg, USMLE World and AMBOSS). In this context, the utilization rate of 61% is quite high and supports the merits of offering a free faculty-reviewed question bank. It is consistent with a prior study that found question banks to be the top-ranked medical student resource for revision of previously learned content [5].

Overall, medical students utilized the platform, although in different settings and with different devices than hypothesized. Perceived utility did not significantly differ across the seven clerkship sites, suggesting that the results were not biased by any one site. In part, the platform was built to support smartphone-based use in response to previous feedback from students who reported that they wished to access question banks on their phones during their commute and when in the hospital. Although the platform was designed for smartphone use to facilitate learning at all times and settings, students mainly accessed the platform via its web version on their laptops at home. These results offer implications for educators in terms of implementing e-learning tools, understanding medical student use of these tools, and assessing their impact. Currently, the Psy-Q platform is optimized for smartphone web use but is not a native app, and an important next step is to explore whether further optimization of the technology will improve utility.

Students were willing to rate questions, with website data recording over 1000 votes on questions. This feedback from students offers a means of quality control and curation of questions, which is a unique strength of web-based learning platforms. Educators could use the Psy-Q platform in the future to understand what types of questions students find useful, as well as access reports on which topics may require more attention, based on the percentages of correct and incorrect answers.

The lack of utilization of a collaborative feature to write questions with educator feedback highlights one challenge for the platform. Although a total of 5535 questions were taken, only two were added by students. This is unfortunately consistent with a prior study that found low acceptability of multiple-choice question writing among students, despite evidence that the task did promote deeper learning [6]. Although our study is not designed to assess the reasons for the low use of this feature, we believe that further training in best practices for writing quality multiple-choice questions and a more extended introduction to the platform for both students and educators may be necessary. This would also offer benefits, as it would address the top reported area for improvement (students reported wanting questions of high quality). Although this study was not designed as an implementation study, the importance of such a study as the next step is clear. Future work should also clarify whether students who take more questions outperform their peers in shelf exams and other objective measures of knowledge (eg, oral exams). In the absence of this information, it is difficult to interpret the relevance of the platform’s 89% satisfaction rate. Objective data could support more structured implementation of the Psy-Q platform in routine clerkship learning (eg, directors assigning a specific number of questions to be completed or written per week).

The finding that more questions were taken according to platform data than reported on the survey suggests that other learners are likely accessing and using the Psy-Q platform. Given that the project’s goal was to create a free, open, and accessible learning tool, we are excited about such use, although our study was not designed to explore the identities of these additional users or their motivation for engaging with the platform. Conceivably, residents or students could revisit the website when preparing for USMLE Step I-III and PRITE examinations, which we were unable to measure in the context of this survey design. The finding that the average user spent over 30 minutes on the platform suggests that students are finding value in this resource. Of note, given that busy faculty are unlikely to receive adequate incentives to write and curate questions, training and then engaging senior medical students and residents as near peer mentors for question writing could be useful in creating a model with long-term sustainability. Thus, the new partnership with the Academy of Consultation Liaison Psychiatry has been helpful in engaging both faculty and trainee members in question writing, and there are plans for additional partnerships with organizations and student groups such as PsychSIGN.

Although our study featured variable site response rates and an inability to directly link student self-reports of the Psy-Q platform to their actual activities, our results still appear valid and reflect real-world usage. The concordance between students reporting taking a mean of 45 questions and web-based data for all users being 42 suggests good concordance between reported and actual use. Not linking survey results to web-based use also offered the benefit of ensuring user privacy and not needing to track student behavior over the internet, which is an ethically challenging space. Finally, our study offers the unique benefit that the Psy-Q platform remains accessible over the internet and active today, meaning that anyone can replicate our results or use these results to expand or augment their own efforts.

### Conclusions

The Psy-Q platform represents an educator- and learner-created platform to augment psychiatry education and develop relevant accessible resources in the digital sphere. Initial results suggest a bright potential for digital tools in psychiatric education and the potential for academic psychiatry to bring leadership, expertise, and value to new learning modalities.

## Appendix

Multimedia Appendix 1 Survey.
