# Supplementary material for: Medical Student Utilization of a Novel Web-Based Platform (Psy-Q) for Question-Based Learning in Psychiatry: Pilot Questionnaire Study
Source: JMIR Med Educ. 2020 Jul 6;6(2):e18340. doi: 10.2196/18340 (PMC7381020; doi:10.2196/18340)
Supplement: Multimedia Appendix 1 [file mededu_v6i2e18340_app1.docx]

**Please note this survey is anonymous. It is part of a research study in which your participation is voluntary. You do not have to complete this survey. It will have no impact on your clerkship evaluation.**

1. For this psychiatry clerkship, how much of your studying and reading was done using online or digital resources as compared to books / print resources?

0% 10% 20% 30% 40% 50% 60% 70% 80% 90% 100%

1. Did you ever access the psy-q.com website?

YES NO

1. If yes to #2, how many questions do you estimate you took (number)? ____________
2. Did you ever submit a new question to the psy-q.com website?

YES NO

1. From which device did you access psy-q.com the most?

Smartphone Tablet Laptop Desktop

1. From which setting did you access psy-q.com the most?

Home Hospital Commuting Library/Study Space

1. Circle all devices and settings that you ever used to access psy-q.com.

Smartphone Tablet Laptop Desktop

Home Hospital Commuting Library/Study Space

1. Overall, did you find psy-q.com to be an unhelpful or useful resource for your learning?

*1 = Unhelpful, 5= Neutral, 10=Most Helpful* 1 2 3 4 5 6 7 8 9 10

1. Would you recommend using psy-q.com to a colleague?

YES NO

1. Circle any aspects of the site that you were unsatisfied with:

Aesthetics Quality of questions/answers Difficulty of questions Ease of Use

1. Did you feel that there was a core feature or aspect missing from the site?

YES NO

If so, what feature or aspect was missing:
